# Carnitine acetyltransferase deficiency mediates mitochondrial dysfunction‐induced cellular senescence in dermal fibroblasts

**DOI:** 10.1111/acel.14000

**Published:** 2023-10-13

**Authors:** Min Ji Song, Chi‐Hyun Park, Haesoo Kim, Sangbum Han, Si Hyung Lee, Dong Hun Lee, Jin Ho Chung

**Affiliations:** ^1^ Department of Dermatology Seoul National University College of Medicine Seoul Republic of Korea; ^2^ Department of Biomedical Sciences Seoul National University Graduate School Seoul Republic of Korea; ^3^ Institute of Human‐Environment Interface Biology, Medical Research Center, Seoul National University Seoul Republic of Korea; ^4^ Institute on Aging, Seoul National University Seoul Republic of Korea

**Keywords:** carnitine acetyltransferase, cellular senescence, mitochondrial dysfunction, oxidative stress, skin aging

## Abstract

Aging is accompanied by impaired mitochondrial function and accumulation of senescent cells. Mitochondrial dysfunction contributes to senescence by increasing the levels of reactive oxygen species and compromising energy metabolism. Senescent cells secrete a senescence‐associated secretory phenotype (SASP) and stimulate chronic low‐grade inflammation, ultimately inducing inflammaging. Mitochondrial dysfunction and cellular senescence are two closely related hallmarks of aging; however, the key driver genes that link mitochondrial dysfunction and cellular senescence remain unclear. Here, we aimed to elucidate a novel role of carnitine acetyltransferase (CRAT) in the development of mitochondrial dysfunction and cellular senescence in dermal fibroblasts. Transcriptomic analysis of skin tissues from young and aged participants showed significantly decreased CRAT expression in intrinsically aged skin. CRAT downregulation in human dermal fibroblasts recapitulated mitochondrial changes in senescent cells and induced SASP secretion. Specifically, CRAT knockdown caused mitochondrial dysfunction, as indicated by increased oxidative stress, disruption of mitochondrial morphology, and a metabolic shift from oxidative phosphorylation to glycolysis. Mitochondrial damage induced the release of mitochondrial DNA into the cytosol, which activated the cyclic GMP‐AMP synthase (cGAS)‐stimulator of interferon genes (STING) and NF‐ĸB pathways to induce SASPs. Consistently, fibroblast‐specific CRAT‐knockout mice showed increased skin aging phenotypes in vivo, including decreased cell proliferation, increased SASP expression, increased inflammation, and decreased collagen density. Our results suggest that CRAT deficiency contributes to aging by mediating mitochondrial dysfunction‐induced senescence.

## Graphical abstract

Transcriptomic analysis of skin tissues showed significantly decreased CRAT expression in intrinsically aged skin. CRAT downregulation in human dermal fibroblasts induced mitochondrial dysfunction, as indicated by increased oxidative stress, disruption of mitochondrial morphology, and a metabolic shift. Mitochondrial dysfunction activated cGAS‐STING and NF‐kB pathways and increased SASP secretion, inducing cellular senescence.

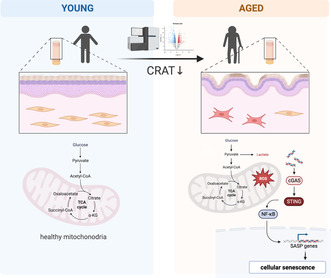

**Abbreviations**
cGAS: cyclic GMP‐AMP synthase
CM: conditioned media
CRAT: carnitine acetyltransferase
DEGs: differentially regulated genes
ECM: extracellular matrix
glycoPER: glycolytic proton efflux rate
GO: Gene ontology
HDFs: human dermal fibroblasts
MMP: matrix metalloproteinase
mtDNA: mitochondrial DNA
NAC: N‐acetylcysteine
OCR: oxygen consumption rate
RNA‐seq: RNA sequencing
ROS: reactive oxygen species
SASP: senescence‐associated secretory phenotype
SA‐β‐gal: senescence‐associated beta‐galactosidase
STING: stimulator of interferon genes

## 1 INTRODUCTION

Aging is a progressive degenerative process involving the accumulation of senescent cells (Campisi, 2013; Jeyapalan & Sedivy, 2008; Lopez‐Otin et al., 2013). Cellular senescence is characterized by cell cycle arrest, telomere shortening, metabolic reprogramming, and secretion of senescence‐associated secretory phenotype (SASP) (Hernandez‐Segura et al., 2018; Kumari & Jat, 2021). The SASP, which includes cytokines, chemokines, proteases, and growth factors (Di et al., 2021), is responsible for causing chronic, low‐grade, and persistent inflammation during aging and is known to mediate various aging‐related conditions such as diabetes, cardiovascular diseases, and skin aging (Franceschi et al., 2018; Spinelli et al., 2020).

Another hallmark of senescent cells is mitochondrial dysfunction (van der Rijt et al., 2020), which is characterized by altered cellular respiration and increased oxidative stress. Since mitochondria are essential for cellular energy production, decreased oxidative phosphorylation and mitochondrial membrane potential as well as increased glycolysis and lactate production are general features of mitochondrial function in senescent cells (Frasca et al., 2021; Mycielska et al., 2022).

Carnitine acetyltransferase (CRAT), is an enzyme mainly localized in the mitochondrial matrix and catalyzes the reversible transfer of acetyl groups between acetyl‐CoA and acetylcarnitine along with other acyltransferases such as carnitine octanoyltransferase and carnitine palmitoyltransferases, CRAT is responsible for mitochondrial acetyl‐CoA balance and regulation of fatty acid oxidation by utilizing short‐ and medium‐chain fatty acids and their corresponding acylcarnitines as substrates (Davies et al., 2016; Melone et al., 2018). Since CRAT regulates acetyl‐CoA level, which is a central source of cellular energy metabolism (Shi & Tu, 2015), it has been extensively studied in muscles with metabolic inflexibility and exercise intolerance, where the active role of mitochondria is indispensable. Muscle‐specific loss of CRAT activity in mice causes disrupted acetyl‐CoA buffering when fed a high‐fat diet or during exercise (Muoio et al., 2012; Seiler et al., 2015). In addition, CRAT deficiency in muscle induces acetyl‐CoA accumulation, which further inhibits glucose utilization by blocking pyruvate dehydrogenase activity (Muoio et al., 2012). The significance of CRAT has been reported in metabolic homeostasis of muscle and brain (Davies et al., 2016; Muoio et al., 2012; Reichenbach et al., 2018; Seiler et al., 2015); however, its role in skin aging remains unexplored.

In this study, we found that CRAT deficiency played a crucial role in mediating mitochondrial dysfunction and cellular senescence in dermal fibroblasts. We determined the morphological and metabolic changes upon CRAT downregulation in the mitochondria and elucidated the effects of CRAT deficiency on cellular senescence and its underlying mechanisms. Our results indicate that CRAT deficiency is a significant driver of mitochondrial dysfunction‐induced cellular senescence.

## 2 MATERIALS AND METHODS

### 2.1 Human skin tissues

Skin samples were obtained from the young (*n* = 6 women, *n* = 6 men; range < 25 years) and elderly (*n* = 6 women, *n* = 6 men; range > 75 years) participants without current or prior skin diseases. The study was conducted in accordance with the principles of the Declaration of Helsinki. All experimental procedures were approved by the Institutional Review Board of Seoul National University Hospital, and written informed consent was obtained from each subject (IRB No. 1410‐134‐621).

### 2.2 RNA‐seq and GO analysis

RNA was isolated from the buttock skin of the young and aged groups (*n* = 12 per group) and subjected to RNA‐seq. RNA quality was assessed by analyzing rRNA band integrity using an Agilent RNA 6000 Nano kit (Agilent Technologies). The constructed cDNA library was sequenced using an Illumina HiSeq2500 (Illumina). Gene expression levels were measured with Cufflinks v2.1.1 (Trapnell et al., 2012) using the gene annotation database Ensembl release 77. Differentially expressed genes were identified using the Cuffdiff tool with default parameter settings and a significance of *p* < 0.05. The GO database classifies genes according to the three categories of biological process (BP), cellular component (CC), and molecular function (MF) and predicts the function of the selected genes. To characterize the genes identified in the DEG analysis, a GO‐based trend test was conducted using Fisher's exact test. *p* < 0.001 were considered statistically significant.

### 2.3 Cell culture

Primary human skin fibroblasts were obtained from healthy donors. The tissue sections were digested with Dispase II (Roche) to remove the epidermis, and then treated with collagenase (Type 1, Worthington Biochemical) and trypsin–EDTA (Thermo Fisher Scientific) to isolate normal HDFs. The cells were subsequently cultured in Dulbecco's modified Eagle medium (DMEM, Welgene) supplemented with 10% fetal bovine serum (FBS, Gibco) and 1% penicillin (Gibco) in a humidified incubator with 5% CO_2_ at 37°C.

### 2.4 Gene silencing by siRNA transfection

HDFs were seeded and transfected with siRNA targeting the negative control siRNA or specific human siRNA using G‐fectin (Genolution) according to the manufacturer's instructions. Negative control siRNA (AccuTarget™ negative control siRNA), and siRNAs targeting CRAT, NF‐ĸB, cGAS, STING, and C/EBPβ were purchased from Bioneer. The siRNA sequences used were as follows:

CRAT forward 5′‐ GAGAAGAUCUGGAACUCAU ‐3′ and reverse 5′‐ AUGAGUUCCAGAUCUUCUC ‐3′; NF‐ĸB forward 5’‐GAUUGAGGAGAAACGUAAA‐3′ and reverse 5’‐UUUACGUUUCUCCUCAAUC‐3′; cGAS forward 5′‐CCUUGUACCCAAGCAUGCA‐3′ and reverse 5′‐UGCAUGCUUGGGUACAAGG‐3′; STING forward 5′‐GAUCAUAAUCACUGCCUUA‐3′ and reverse 5′‐UAAGGCAGUGAUUAUGAUC‐3′; C/EBPβ forward 5′‐ GGCCCUGAGUAAUCGCUUA −3′ and reverse 5′‐ UAAGCGAUUACUCAGGGCC ‐3′.

Single siRNA transfection was conducted at 300 pmol/mL and double siRNA transfection at 150 pmol/mL to equalize the total transfection concentration. After 48 h, the fibroblast culture was washed once with phosphate‐buffered saline (PBS) and replaced with serum‐free DMEM.

### 2.5 RNA extraction and quantitative RT‐PCR

RNA was extracted by homogenization of cells and skin tissues using the RNAiso Plus reagent (Takara Bio). RNA (3 μg) was used to synthesize cDNA using First Strand cDNA Synthesis Kit (Thermo Fisher Scientific) according to the manufacturer's instructions. PCR was performed on a 7500 Real‐time PCR System (Applied Biosystems) using SYBR Premix Ex Taq (Takara Bio), ROX, and primer pairs. The data were analyzed using the comparative ΔΔCt method, normalized to 36B4 expression, and presented as fold changes.

The primers used for RT‐qPCR are as follows:

**Table:** 

Gene	Forward primer sequence	Reverse primer sequence
CRAT	GTCCCTGGACCACTACCTGA	CTTGAGCCACCACTCAGACA
MMP‐1	AAGCGTGTGACAGTAAGCTA	AACCGGACTTCATCTCTG
IL1α	TGTGACTGCCCAAGATGAAG	AAGTTTGGATGGGCAACTGA
IL1β	CTGTCCTGCGTGTTGAAAGA	TTCTGCTTGAGAGGTGCTGA
IL6	GCAGATGAGTACAAAAGTCC	GCAGAATGAGATGAGTTGTC
IL8	CAGGAATTGAATGGGTTTGC	AAACCAAGGCACAGTGGAAC
CXCL1	AGTGGCACTGCTGCTCCT	AGCTTTCCGCCCATTCTT
CXCL2	GGGCAGAAAGCTTGTCTCAA	GCTTCCTCCTTCCTTCTGGT
cGAS	ACGTGCTGTGAAAACAAAGAAG	GTCCCACTGACTGTCTTGAGG
STING	GAGAGCCACCAGAGCACA	TAGATGGACAGCAGCAACAG
p65	GCGAGAGGAGCACAGATACC	CTGATAGCCTGCTCCAGGTC
C/EBPβ	GAGTACAAGATCCGGCGTGA	GGGCAGCTGCTTGAACAAGT
36B4	TCGACAATGGCAGCATCTAC	TGATGCAACAGTTGGGTAGC
mCRAT	CTAACCTCCAACCACCGAAA	CCACCACCATGTAGCATCTG
mGAPDH	GATGCCCCCATGTTTGTG	ACAACCTGGTCCTCAGTG
mCDKN1A	ATTCCATAGGCGTGGGACCT	TCCTGGGCATTTCGGTCAC
mIL6	GCTACCAAACTGGATATAATCAGGA	CCAGGTAGCTATGGTACTCCAGAA
mCXCL1	GCTGGGATTCACCTCAAGAA	TCTCCGTTACTTGGGGACAC
mCXCL2	AGTGAACTGCGCTGTCAATG	TCCAGGTCAGTTAGCCTTGC
mCXCL9	GGAACCCTAGTGATAAGGAATGCA	TGAGGTCTTTGAGGGATTTGTAGTG
mCXCL11	CCGAGTAACGGCTGCGACAAAG	CCTGCATTATGAGGCGAGCTTG

### 2.6 Western blotting

Skin tissue samples and cultured cells were homogenized and lysed in radioimmunoprecipitation (RIPA) buffer (Merck) containing protease inhibitor mixture (Roche Applied Science) and phosphatase inhibitor mixture (Sigma‐Aldrich). Tissue extracts and cell lysates were centrifuged at 12,000 rpm, 4°C for 20 min, and supernatants were collected for western blot analysis. The protein concentration of the samples was determined using Bradford reagent (Bio‐Rad Laboratories). Equal concentrations of the protein extracts were loaded onto 8% or 12% sodium dodecyl sulfate (SDS)‐polyacrylamide gels, separated electrophoretically, and transferred to polyvinylidene fluoride (PVDF) membranes (Roche Applied Science). The membranes were blocked with 5% skim milk for 1 h at room temperature with agitation, followed by incubation with primary antibodies against human CRAT (NBP1‐86616, Novus), t‐p65 (#8242, CST), p‐IĸBα (#9246, CST), t‐IĸBα (#9242, CST), α‐tubulin (sc23948, Santa Cruz), PDHE1α (sc377092, Santa Cruz), Lamin B1 (sc374015, Santa Cruz), GAPDH (1:1000, CSB‐PA00029A0Rb, Cusabio, Houston, TX, USA), and β‐actin (MA5‐15739, Invitrogen) at 4°C overnight, shaking. The membrane was washed three times with Tris‐buffered saline with 0.1% Tween 20 detergent (TBST) and incubated with secondary antibody for 1 h at room temperature. Horseradish peroxidase‐conjugated anti‐goat, anti‐rabbit, and anti‐mouse IgG (Genetex) were used as secondary antibodies. The bands were visualized using an enhanced chemiluminescence detection system (Thermo Fisher Scientific). The signal intensity was measured using ImageJ software (National Institutes of Health, Bethesda, MD, USA).

### 2.7 SA‐β‐gal staining

The SA‐β‐gal staining kit (#9860, Cell Signaling Technology, Danvers, MA, USA) was used according to the manufacturer instructions. Briefly, cells were fixed in a fixative solution for 15 min, then incubated in β‐galactosidase staining solution (pH 6.0) at 37°C overnight in a dry incubator without CO_2_. The blue SA‐β‐Gal‐positive cells were imaged and counted under a bright field microscope. For cells subjected to siRNA‐mediated knockdown, the medium was replaced with 10% DMEM 2 days after transfection, and SA‐β‐gal staining was performed 8 days following this medium change.

### 2.8 Cytokine analysis

Cytokines secreted in supernatants were determined by a magnetic bead‐based multiplex assay using Bio‐Plex® multiplex system (Bio‐Rad Laboratories) according to the manufacturers' instructions. The concentrations of multiple cytokines were measured using Bio‐Plex Pro Human Cytokine IL‐1β Set (#171B5001M, Bio‐Rad), Bio‐Plex Pro Human Cytokine IL‐6 Set (#171B5006M, Bio‐Rad), and Bio‐Plex Pro Human Cytokine IL‐8 Set (#171B5008M, Bio‐Rad).

### 2.9 Cell viability assay

The cell viability was assessed using a Cell Counting Kit‐8 (CCK8; #CK04, Dojindo) according to the manufacturers' protocol. Briefly, CCK8 solution was added and incubated for 40 min in a humidified incubator with 5% CO_2_ at 37°C. The absorbance was measured at 450 nm using a microplate reader (VersaMax, Molecular Devices Corporation), and all samples were measured in triplicate.

### 2.10 Immunofluorescence staining of human and mouse skin tissues

Immunofluorescence analysis of paraffin‐embedded sections was performed using human and mouse skin tissue samples. Paraffin‐embedded skin tissue sections were deparaffinized and rehydrated using ethanol series. The sections were subjected to heat‐induced antigen retrieval in 0.01 M citrate buffer (pH 6.0). After blocking with a blocking solution for 30 min at room temperature, the sections were incubated with primary antibody against CRAT (GTX103000, Genetex), p16 (ab189034, Abcam) and vimentin (MAB2105, R&D Systems), overnight at 4°C, and then labeled with the fluorescent secondary antibody, Alexa Fluor 488 (A‐11006, Invitrogen) or Alexa Fluor 594 (A‐11012, Invitrogen) after washing. Nuclei were counterstained with 4′,6‐diamidino‐2‐phenylindole (DAPI) and the sections were mounted in Faramount aqueous mounting medium (Dako). The sections were imaged using a confocal microscope (Leica STED CW; Leica Microsystems).

### 2.11 Luciferase reporter assay

HDFs were transfected with pGL3 basic reporter vector (Promega) or pGL3‐NF‐ĸB‐luc vector which contains human NF‐ĸB promoter and the firefly luciferase gene. The cells were transiently transfected with pGL3 basic reporter or pGL3‐NF‐ĸB‐luc vector along with the pRL‐TK, which served as an internal control for transfection efficiency using Lipofectamine 3000.

### 2.12 Subcellular fractionation

Nuclear and cytoplasmic fractionation was conducted using NE‐PER™ Nuclear and Cytoplasmic Extraction Reagents (Thermo Fisher Scientific), and cytosolic, mitochondrial, and nuclear fractions were obtained using Cell Fractionation Kit (ab109719, Abcam), according to the manufacturer's protocol. The cytosolic fraction was subsequently subjected to genomic DNA extraction using the DNeasy Blood & Tissue Kit (Qiagen) according to the manufacturer's protocol.

### 2.13 Measurement of mitochondrial ROS levels and morphologies by live cell imaging

Mitochondrial ROS levels were measured using the MitoSOX™ Red mitochondrial superoxide indicator (Invitrogen) according to the manufacturer's protocol. Briefly, cells were incubated with 2.5 μM MitoSOX™ Red for 20 min and then washed with PBS three times. Live cells were imaged using a confocal microscope (Leica STED CW). The intensity of red fluorescence was analyzed using ImageJ software. The corrected total cell fluorescence (CTCF) was calculated according to the formula: CTCF = Integrated Density − (Area of selected cell X Mean fluorescence of background reading).

The mitochondrial skeletal length, area, and number were calculated using the Operetta CLS high‐content analysis system (Perkin Elmer). HDFs were seeded in 96‐well dishes, and stained with MitoTracker™ Green FM for 30 min (Invitrogen) and Hoechst 33342 for 15 min (Thermo Fisher Scientific).

### 2.14 Mitochondrial energy metabolism analysis

The mitochondrial respiration and metabolic profiles of the dermal fibroblasts were determined using a Seahorse XF96 Extracellular Flux Bioanalyzer (Seahorse Bioscience) to measure the basal and maximal OCR and extracellular acidification rate (ECAR), as suggested by the manufacturer. Briefly, HDFs were seeded in 96‐well microplates containing an XF96 Extracellular Flux analyzer with four blank background wells and incubated overnight. After equilibration for 4 h in a non‐CO_2_ incubator prior to the assay, a 96‐well microplate was used for analysis.

For Mito Stress test, the cells were analyzed at basal conditions after injection of 2 μM oligomycin to inhibit ATP synthase, 1 μM carbonyl cyanide 4‐trifluoromethoxy‐phenylhydrazone (FCCP) to induce maximal respiration, and 0.5 μM rotenone and antimycin A to inhibit complexes I and III. Basal respiration, maximal respiration, and spare respiratory capacity were calculated. For glycolytic rate test, the cells were analyzed following injections with 0.5 μM rotenone/antimycin A to inhibit mitochondrial respiration and 50 μM 2‐deoxyglucose (2‐DG) to inhibit glycolysis. PER, glycoPER, basal glycolysis, basal proton efflux rate, and compensatory glycolysis were also calculated. All mitochondrial complex inhibitors were purchased from Seahorse Bioscience.

### 2.15 Fibroblast‐specific CRAT‐knockout mouse

The CRAT^flox/flox^ mice were generously provided by Dr. Randall Mynatt (Pennington Biomedical Research Center, Baton Rouge, LA, USA) (Muoio et al., 2012). Fibroblast‐specific CRAT‐knockout mice were generated by crossing B6.Cg‐Tg(Col1a2‐cre/ERT,‐ALPP)7Cpd/J (Col1a2^cre^; Jackson Laboratory, #029567) mice with CRAT^flox/flox^ mice. Cre‐negative CRAT^flox/flox^ were used as controls in all the experiments. Skin tissues were either frozen in liquid nitrogen and stored at −80°C for RNA analysis or fixed in 4% formaldehyde for immunohistochemistry and immunofluorescence staining. The dermis of skin tissues was used to culture primary fibroblasts for analyzing the effects of CRAT knockout.

All animal procedures were approved by the Institutional Animal Care and Use Committee of the Seoul National University Hospital (IACUC No. 20‐0105‐S1A0).

### 2.16 Masson's trichrome staining

Masson's trichrome staining was performed on paraffin‐embedded mouse back‐skin sections using standard protocols. Dermal thickness and collagen density were quantified using the ImageJ software.

### 2.17 Statistical analysis

GraphPad Prism 9.0 software was used for all statistical analytical procedures.

## 3 RESULTS

### 3.1 CRAT is significantly downregulated in aged human skin in vivo

**FIGURE 1:**
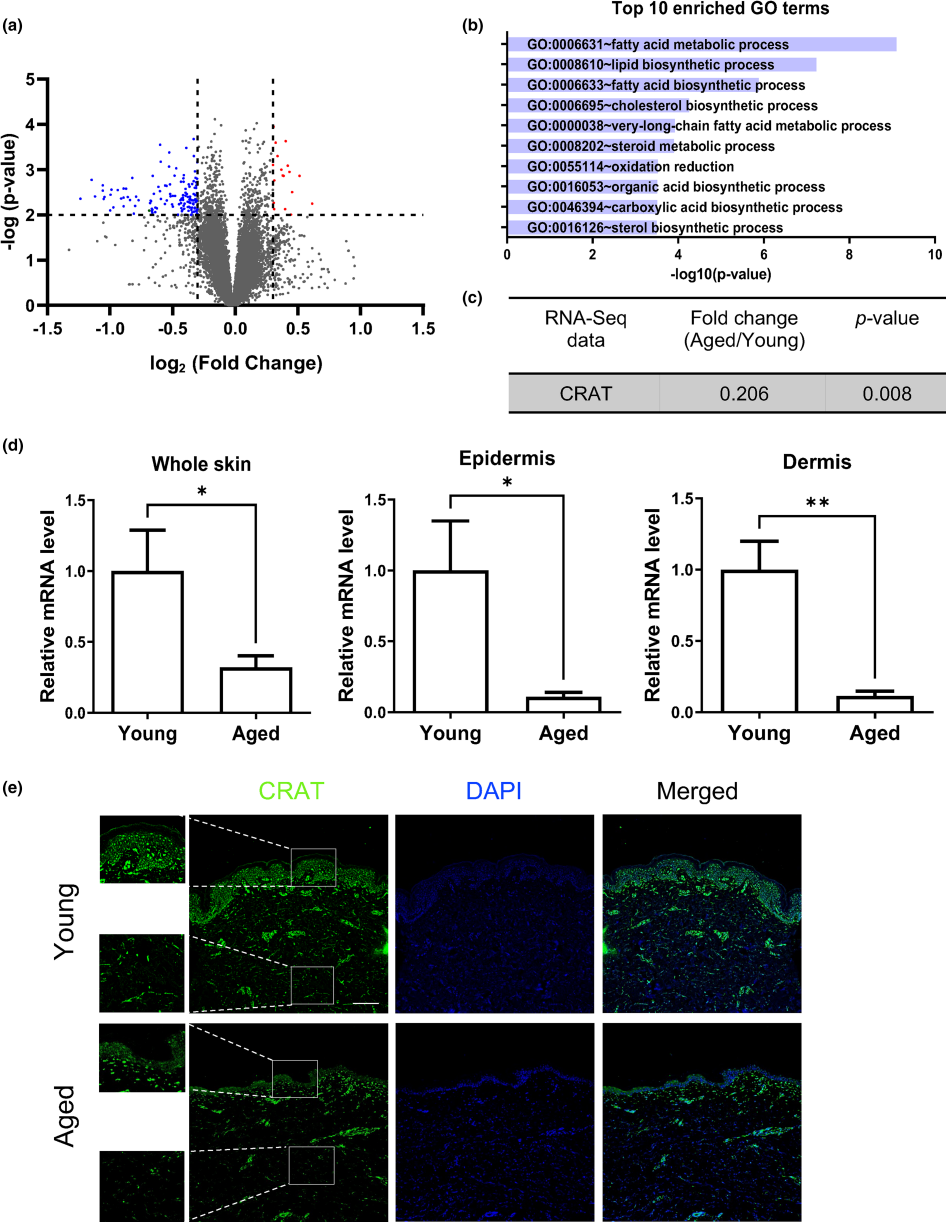
Carnitine acetyltransferase (CRAT) is downregulated in aged human skin in vivo. (a) Differentially expressed genes (DEGs) analyzed by RNA sequencing (RNA‐seq) analysis of the skin of the aged (age > 75 years, *n* = 12) and young (age < 25 years, *n* = 12) participants are displayed on the volcano plot. (b) Gene ontology (GO) analysis revealed an enrichment of DEGs in the aged compared to the young groups. To characterize the identified genes from DEG analysis, GO‐based trend test was performed using Fisher's exact test. *p* < 0.001 was considered statistically significant. (c) Fold change and *p*‐value of CRAT expression differences in aged/young groups from RNA‐seq data. (d) Validation of *CRAT* mRNA expression levels by real time‐PCR using whole skin, epidermis, and dermis samples (*n* = 8 for whole skin, *n* = 4 for epidermis and dermis). Data represent the mean ± standard error of mean (SEM). **p < 0.01, *p < 0.05 versus young analyzed by *t* test. (e) Immunofluorescence staining of CRAT and nuclei staining using 4′,6‐diamidino‐2‐phenylindole (DAPI) in paraffin‐embedded skin tissue sections from the young (*n* = 3) and aged (*n* = 3) donors. Representative images are shown. *Scale bar* = 100 μm.

To identify the novel gene governing intrinsic skin aging, we performed RNA sequencing (RNA‐seq) analyses using human buttock (sun‐protected) skin obtained from young (*n* = 12) and aged individuals (*n* = 12) (Figure 1a). Gene ontology (GO) analysis using differentially regulated genes (DEGs) revealed that fatty acid metabolic process was the most significantly enriched GO term (Figure 1b). Among the genes associated with fatty acid metabolism, CRAT was the gene whose expression level was not only relatively high in skin tissue, but also significantly decreased in the aged skin by approximately 80% in the RNA‐seq data (Figure 1c). Substantial downregulation of *CRAT* mRNA level in aged skin was validated in whole skin tissues, epidermis, and dermis using quantitative real‐time PCR (Figure 1d). Immunofluorescent staining analysis showed that CRAT was expressed in most cells in the skin, and its expression was reduced in both the epidermis and dermis of aged skin compared to young skin in vivo (Figure 1e).

Skin aging‐related pathologies include the degradation of extracellular matrix (ECM), uncompensated oxidative stress, and the accumulation of senescent cells, which mainly occur in the dermis. Increasing evidence suggests that senescent fibroblasts in aged skin produce SASPs and contribute to inflammaging (Pilkington et al., 2021; Wlaschek et al., 2021). The expression level of CRAT was relatively high in human dermal fibroblasts (HDFs) compared to epidermal keratinocytes by approximately tenfold (Figure S1a). The mRNA level of CRAT was significantly decreased in primary cultured fibroblasts from aged individuals compared to those from the young (Figure S1b). Thus, we focused on the mechanistic role of CRAT on aging in primary dermal fibroblasts.

### 3.2 CRAT downregulation recapitulated cellular senescence and induced SASPs in human dermal fibroblasts

**FIGURE 2:**
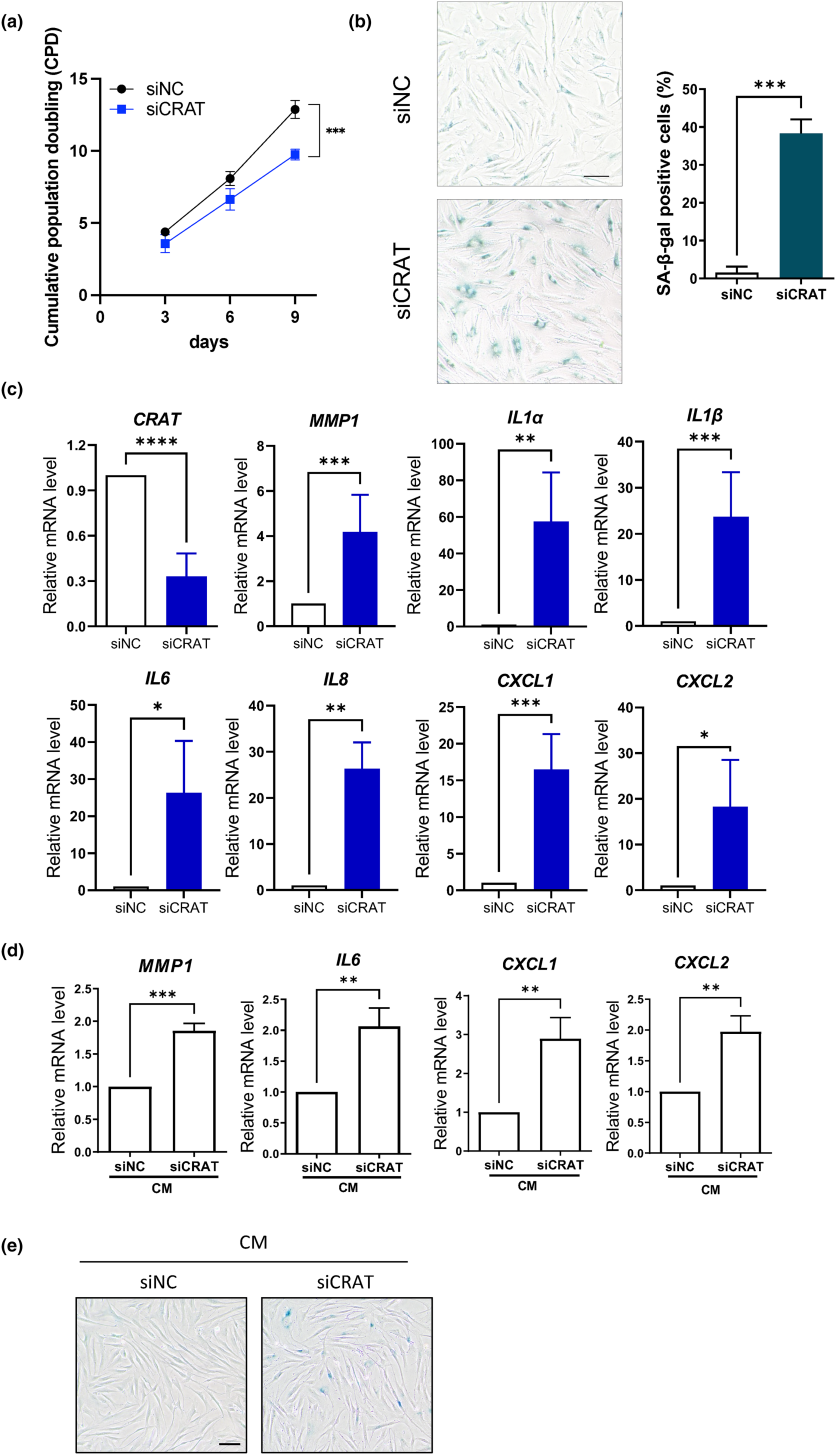
Carnitine acetyltransferase (CRAT) knockdown induces cellular senescence and regulates senescence‐associated secretory phenotype (SASP). (a) Cumulative population doublings were determined in human dermal fibroblasts (HDFs) cultured for 3, 6, and 9 days after transfection with siRNA targeting either negative control siRNA (siNC) or CRAT (siCRAT). (b) HDFs transfected with siNC and siCRAT were subjected to senescence‐associated‐β‐galactosidase (SA‐β‐gal) staining 10 days after transfection. Percentage of SA‐β‐gal‐positive cells was quantified (*n* = 3) and representative photos are shown. *Scale bar* = 100 μm. (c) The mRNA levels of SASP genes were determined by quantitative real‐time PCR (RT‐PCR) after siNC and siCRAT transfection for 48 h followed by 72 h incubation after media change (*n* = 5). (d) mRNA expression levels of SASPs of normal fibroblasts cultured for 5 days in conditioned media (CM) from CRAT‐knockdown cells collected 5 days after transfection (*n* = 3). (e) HDFs were cultured for 10 days in the conditioned media (CM) from CRAT‐knockdown cells, mixed with 10% DMEM in a 1:1 ratio, and then subjected to SA‐β‐gal staining (*n* = 3). *Scale bar* = 100 μm. Data are shown as mean ± standard error of mean (SEM). ***p < 0.001, **p < 0.01, *p < 0.05, versus siNC analyzed by *t* test.

To delineate the role of CRAT in skin aging, we first examined CRAT downregulation induced cellular senescence in HDFs. CRAT knockdown significantly decreased the cell proliferation rate, as determined by the decreased number of cells 5 days after transfection (Figure 2a). Along with reduced cell proliferation, CRAT silencing increased the number of senescent cells as determined by the increased number of senescence‐associated beta‐galactosidase (SA‐β‐gal)‐positive cells (Figure 2b). Moreover, the mRNA expression levels of SASP factors, such as matrix metalloproteinase (*MMP*)*1*, *IL1α*, *IL1β*, *IL6*, *IL8*, *CXCL1*, and *CXCL2*, were significantly increased after knockdown of CRAT (Figure 2c). The protein levels of secreted SASPs, such as *IL1β*, *IL6*, and *IL8*, were also confirmed through cytokine analysis (Figure S2a). As three different CRAT siRNA sequences showed comparable knockdown efficacy, decreased cell proliferation rate, and increased SASP mRNA expression levels (data not shown), one representative siRNA was used in further experiments.

Senescent cells are known to affect adjacent cells via SASPs in a paracrine manner, thereby contributing to aging (Kumari & Jat, 2021). To examine the paracrine effects of CRAT downregulation, HDFs from young donors were cultured in conditioned media from fibroblasts treated with CRAT or scrambled siRNAs. The conditioned media from CRAT‐knockdown fibroblasts were sufficient to induce increased the mRNA expression levels of inflammatory cytokines and chemokines, such as *MMP1*, *IL6*, *CXCL1*, and *CXCL2* (Figure 2d), as well as an increase in SA‐β‐gal‐positive cells (Figure 2e). These results suggest that CRAT‐knockdown cells became senescent and subsequently exerted detrimental effects on neighboring cells in a paracrine manner by secreting SASPs.

### 3.3 CRAT downregulation induced oxidative stress and mitochondrial damage

**FIGURE 3:**
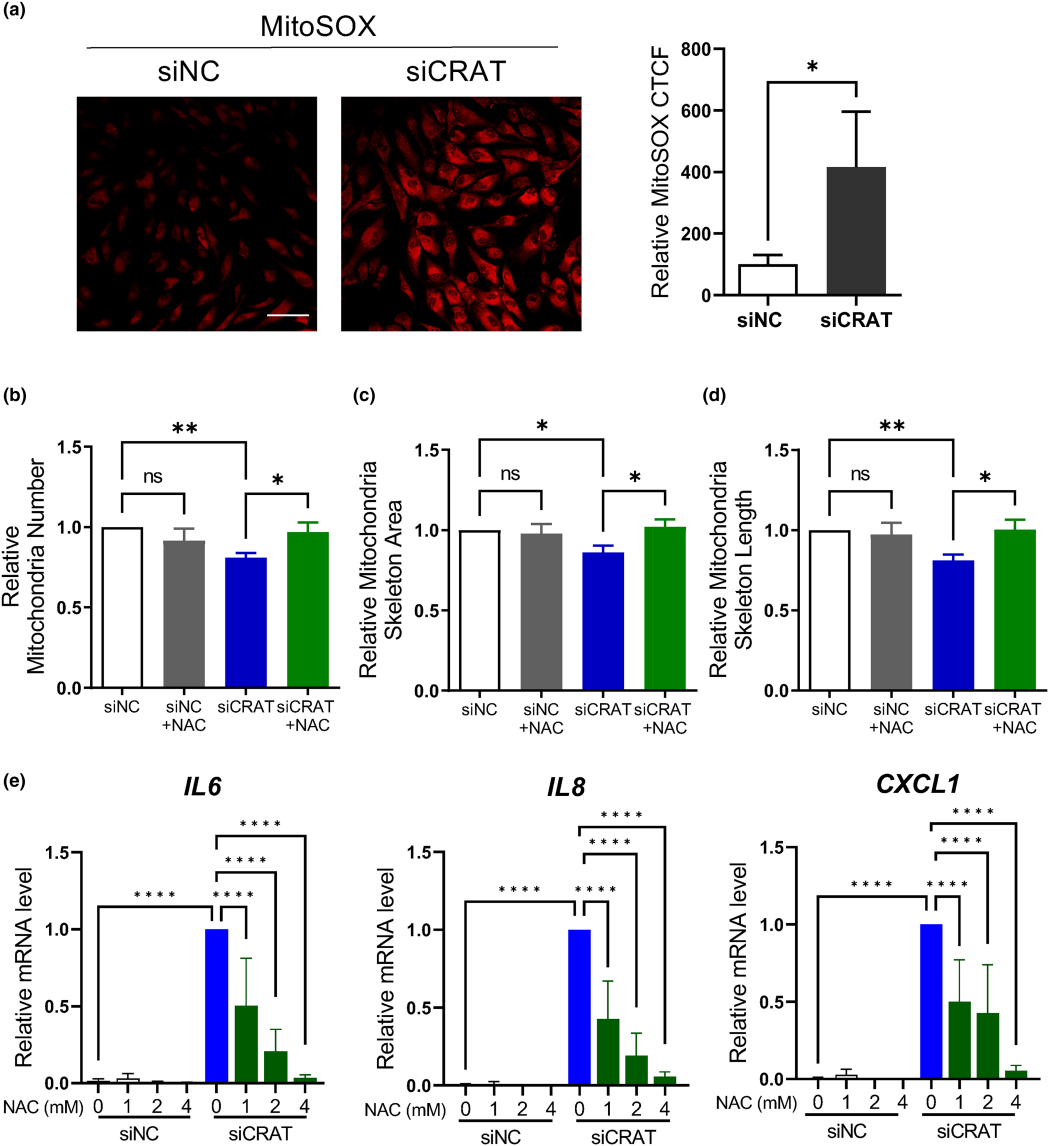
Carnitine acetyltransferase (CRAT) downregulation induces oxidative stress and mitochondrial dysfunction. (a) For detection of mitochondrial reactive oxygen species (ROS), siNC‐ and siCRAT‐transfected cells were stained with MitoSOX™ 2 days after transfection and then imaged using confocal microscopy. *Scale bar* = 100 μm. The image is representative of three independent experiments. The mitochondrial ROS level was quantified using ImageJ by calculating fluorescence intensity of 10 cells per image. A relative corrected total cell fluorescence (CTCF) was calculated in relation to an average CTCF of siNC cells. For quantitative imaging of (b) mitochondrial number, (c) skeleton area, and (d) skeleton length, human dermal fibroblasts (HDFs) were transfected with siNC or siCRAT with or without *N*‐acetylcysteine (NAC) (2 mM) for 48 h. The cells were cultured in 10% Dulbecco's modified Eagle medium (DMEM) for 2 days, and then seeded in a 96‐well plate 12 h prior to Operetta CLS analysis (*n* = 3). The cells were stained using MitoTracker™ Green for quantification. (e) HDFs were transfected with siRNAs in the presence or absence of NAC at the indicated concentrations for 48 h. The cells were harvested 5 days after transfection (*n* = 4). Senescence‐associated secretory phenotype (SASP) genes at the mRNA level were determined by quantitative real‐time PCR. Data are shown as mean ± standard error of mean (SEM). ****p < 0.0001, ***p < 0.001, **p < 0.01, *p < 0.05 versus siNC or siCRAT analyzed by *t* test (a) or analysis of variance (ANOVA) (b–e).

CRAT is primarily located in the mitochondria and cellular senescence is associated with alterations in mitochondrial function due to oxidative stress (Passos et al., 2010). Hence, we determined whether CRAT downregulation induces reactive oxygen species (ROS) accumulation in the mitochondria. CRAT knockdown significantly increased mitochondrial ROS levels, as visualized by MitoSOX fluorescence staining, which measures mitochondria‐specific ROS production (Figure 3a). Morphological changes in the mitochondria were examined by quantitative measurements of mitochondrial number, skeletal area, and skeletal length. CRAT‐knockdown cells showed a significant reduction in the mitochondrial number (Figure 3b), mitochondrial skeletal area (Figure 3c), and skeletal length (Figure 3d). These changes are most likely attributable to the increased mitochondrial disruption induced by ROS (Panusatid et al., 2022). To confirm the role of ROS in mitochondrial damage following CRAT knockdown, we used the ROS scavenger, *N*‐acetylcysteine (NAC). Indeed, NAC treatment rescued the reduction in mitochondrial number (Figure 3b) and skeletal area (Figure 3c) and skeletal length (Figure 3d). The cell viability after NAC treatment remained relatively stable at a concentration of 2 mM (Figure S3a), and the protein expression of CRAT was unchanged at 2 mM of NAC (Figure S3b). Therefore, we used 2 mM of NAC to assess changes in mitochondrial morphology. Moreover, NAC significantly reversed CRAT‐induced senescence phenotypes, such as SASP production, including *IL6*, *IL8*, *CXCL1*, and *MMP1* (Figure 3e), and an increase in SA‐β‐gal‐positive cells (Figure S3c), suggesting that ROS induced by CRAT knockdown contributes to mitochondrial damage and SASP upregulation.

### 3.4 CRAT knockdown disrupted mitochondrial energy homeostasis

**FIGURE 4:**
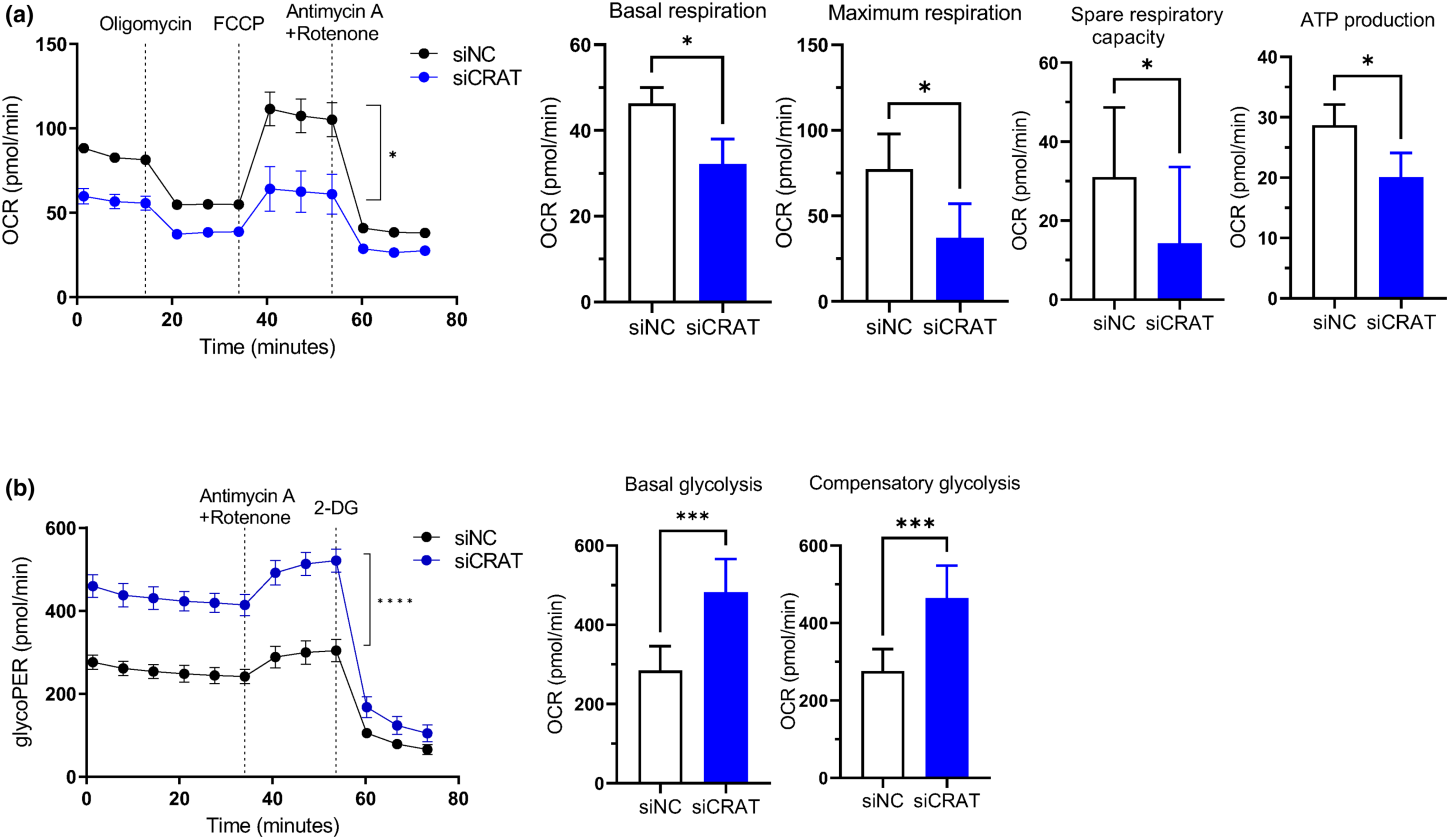
Carnitine acetyltransferase (CRAT) disturbs energy metabolism within mitochondria. Human dermal fibroblasts (HDFs) were transfected with siRNAs targeting NC or CRAT for 48 h and reseeded at approximately 3 × 10^5^ cells/well in a 96‐well microplate 12 h prior to analysis. Absorbance was recorded using the Seahorse XF96 analyzer twelve times at 6 min intervals at baseline following injections of the indicated inhibitors. (a) The Mito Stress test was used to measure the oxygen consumption rate (OCR) (*n* = 4). Inhibitors were injected at indicated time points: 2 μM oligomycin to inhibit ATP synthase, 1 μM carbonyl cyanide 4‐trifluoromethoxy‐phenylhydrazone (FCCP) to induce maximal respiration, and 0.5 μM rotenone (Rot) and antimycin A (AA) to inhibit complex I and III. Basal respiration, maximal respiration and spare respiratory capacity were calculated using the following formulae: basal respiration = measurement before the first injection–non‐mitochondrial respiration rate; maximal respiration = maximum rate measurement after FCCP injection – non‐mitochondrial respiration; spare respiration capacity = maximal respiration–basal respiration; ATP production = measurement before oligomycin treatment–minimum rate measurement after oligomycin injection. (b) Glycolytic rate test was used to measure glycolytic proton efflux rate (glycoPER) (*n* = 3). For glycolytic rate test, cells were analyzed following injections with 0.5 μM Rot/AA to inhibit mitochondrial respiration and 50 μM 2‐deoxyglucose (2‐DG) to inhibit glycolysis. Basal and compensatory glycolysis were calculated using the following formulae: basal glycolysis = measurement before the first injection and compensatory glycolysis = measurement before the second injection. Data are shown as mean ± standard error of mean (SEM). ****p < 0.0001, ***p < 0.001, *p < 0.05 versus siNC analyzed by *t* test (a, b).

Given the central role of mitochondria in energy metabolism, we focused on metabolic changes driven by CRAT silencing. To examine the mitochondrial energy metabolism in HDFs treated with CRAT siRNA, mitochondrial oxidative phosphorylation, and glycolysis were measured by oxygen consumption rate (OCR) and glycolytic proton efflux rate (glycoPER), respectively (Figure 4a,b). Notably, basal respiration, maximum respiration, and spare respiratory capacity were significantly decreased after CRAT knockdown (Figure 4a), suggesting that oxidative phosphorylation in the mitochondria was impaired after CRAT silencing. Mitochondrial ATP production was also decreased, most likely due to mitochondrial damage (Figure 4a). In contrast, glycolysis, as measured by glycoPER, was increased in CRAT‐knockdown fibroblasts (Figure 4b). Basal glycolysis and compensatory glycolysis rate were both significantly increased (Figure 4b), implying that glucose utilization was more prone to lactate production than entry into the mitochondria for oxidative phosphorylation. CRAT knockdown induced a metabolic switch from oxidative phosphorylation to glycolysis in the mitochondria, suggesting that non‐mitochondrial respiration was increased instead of mitochondrial respiration, as a compensatory mechanism for insufficient energy production in damaged mitochondria. These results suggest that increased mitochondrial oxidative stress in CRAT‐knockdown fibroblasts resulted in mitochondrial damage, which subsequently affected energy metabolism, characterized by reduced mitochondrial oxidative phosphorylation and increased glycolysis.

### 3.5 CRAT knockdown released mitochondrial DNA (mtDNA) into the cytosol, resulting in activation of cGAS‐STING and NF‐kB pathway and SASP induction

**FIGURE 5:**
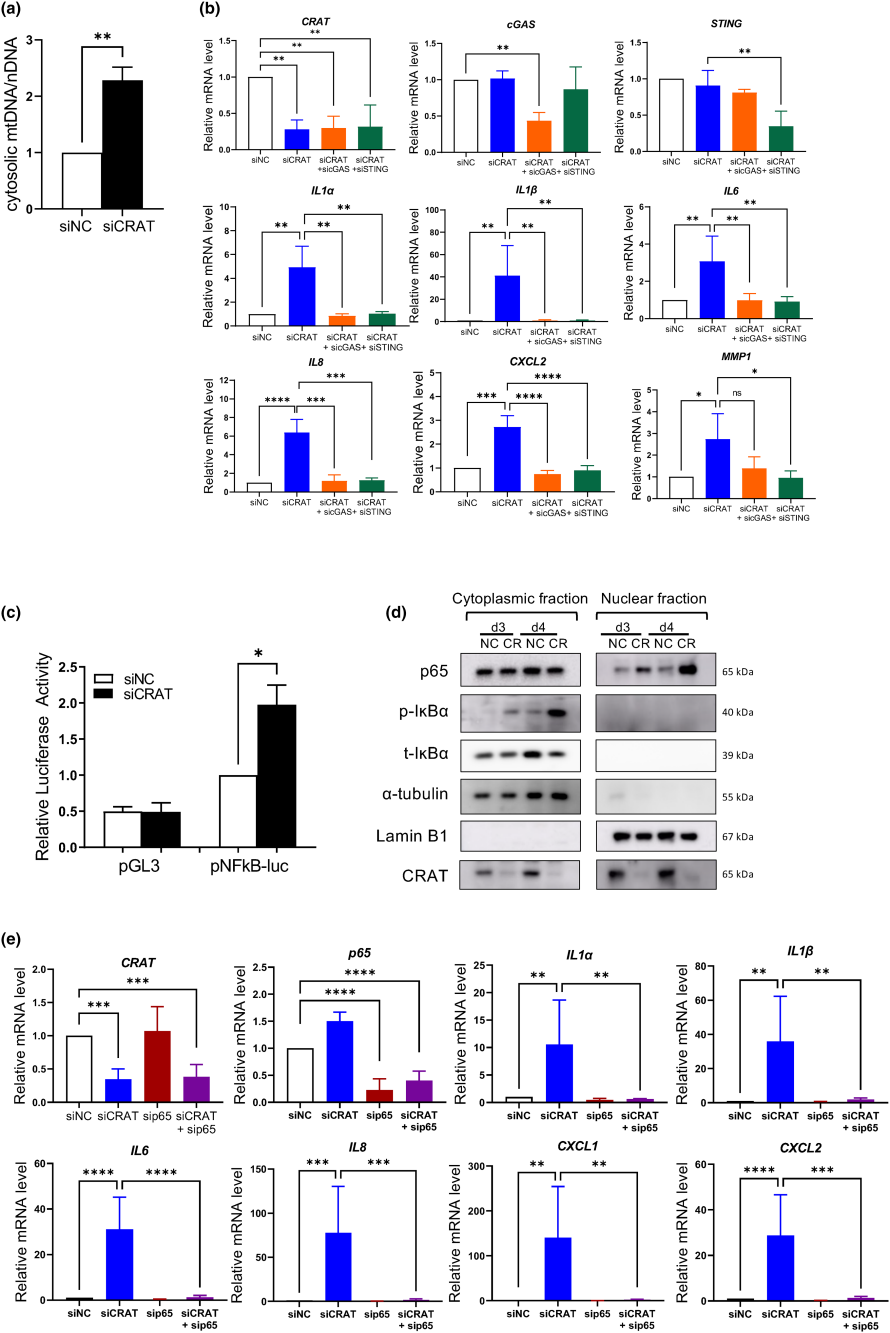
Carnitine acetyltransferase (CRAT) knockdown releases mitochondrial DNA (mtDNA) into cytosol and activates cGAS‐STING and NF‐ĸB pathways. (a) Cytosolic mtDNA level was measured by RT‐PCR. Human dermal fibroblasts (HDFs) were transiently transfected with siNC or siCRAT for 48 h and the cells were subjected to cellular fractionation to separate cytosolic fraction by removing nuclear and mitochondrial fractions. Genomic DNA in the cytosol of CRAT‐knockdown fibroblasts were analyzed to measure the cytosolic mtDNA. mtDNA level was normalized to nDNA level (*n* = 3). (b) The mRNA expression levels of senescence‐associated secretory phenotype (SASP) genes were measured by RT‐PCR after co‐transfection of siNC or siCRAT without or with either sicGAS or siSTING. Media was changed 48 h after transfection and cells were analyzed 3 days after media change (*n* = 3). (c) The activation of NF‐ĸB was measured by luciferase reporter assay. HDFs were transfected with empty pGL3 vector and pNF‐ĸB‐luc vector 2 days after transfection with siNC or siCRAT. Data were normalized to Renilla luciferase activity (*n* = 3). (d) Accumulation of p65 in the nucleus and phosphorylation of p‐IĸBα were detected by western blotting after separation of nuclear and cytoplasmic fractions. Cells were harvested at day 3 (d3) and day 4 (d4) after transfection with siNC (NC) or siCRAT (CR), followed by nuclear and cytoplasmic fractionation. The purity of cytoplasmic and nuclear fractions was determined using α‐tubulin and Lamin B1, respectively. The representative western blot of three independent experiments is shown. (e) HDFs were subjected to transient transfection for 48 h with siNC, siCRAT, or sip65 or co‐transfection of siCRAT and sip65. mRNA expression of SASP genes were analyzed by RT‐PCR 3 days after media change (*n* = 4). Data are shown as mean ± standard error of mean (SEM), ****p < 0.0001, ***p < 0.001, **p < 0.01, *p < 0.05 versus siNC or siCRAT analyzed by *t* test (a, c) or analysis of variance (ANOVA) (b, d).

These results raise questions regarding the molecular mechanisms connecting mitochondrial dysfunction and SASPs upon CRAT downregulation. Upon mitochondrial damage, mtDNA released into the cytosol may serve as a damage‐associated molecular pattern (DAMP) to activate inflammatory pathways and SASP genes (Riley & Tait, 2020; Takahashi et al., 2018). Thus, the release of mtDNA in CRAT‐knockdown fibroblasts was measured by performing mtDNA‐specific PCR of the isolated cytosolic fraction after the elimination of mitochondria and nucleus. The mtDNA‐to‐nuclear DNA (18s) ratio was significantly increased in the cytosol after CRAT knockdown (Figure 5a), implying that CRAT knockdown induced the release of mtDNA into the cytosol due to mitochondrial damage.

Cytosolic mtDNA is sensed through the cyclic GMP‐AMP synthase (cGAS), leading to the production of second messenger 2′3′ cyclic GMP‐AMP (cGAMP), which serves as an agonist of stimulator of interferon genes (STING) (Dou et al., 2017; Hu et al., 2022). The cGAS‐STING pathway is an important cytosolic DNA sensor system for innate immunity and recent studies have implicated the importance of cGAS in cellular senescence (Dou et al., 2017; Gluck et al., 2017; Hu et al., 2022; Yang et al., 2017). To examine whether the cGAS‐STING pathway mediates CRAT silencing‐induced SASP regulation, we silenced cGAS or STING using siRNA and found that knockdown of either cGAS or STING led to the inhibition of CRAT siRNA transfection‐induced SASPs, including *IL1α*, *IL1β*, *IL6*, *IL8*, *CXCL2*, and *MMP1* (Figure 5b; Figure S4). Secretion of SASPs and the increase in SA‐β‐gal‐positive cells were also inhibited by the knockdown of cGAS and STING (Figure S5a,b). Taken together, the cytosolic mtDNA released because of mitochondrial damage in CRAT‐knockdown fibroblasts triggered the cGAS‐STING pathway, thereby leading to the production of SASPs.

Because cGAS‐STING is known to activate SASPs via NF‐ĸB (Gao et al., 2020), which is considered a master regulator of SASPs (Songkiatisak et al., 2022), we next explored the role of NF‐ĸB in CRAT downregulation‐induced SASPs. The NF‐ĸB activity, measured by luciferase reporter assay after transfection of pNF‐ĸB plasmid, was increased after CRAT knockdown (Figure 5c).

The activation of NF‐ĸB was further confirmed by its translocation from the cytoplasm to the nucleus, as observed by increased p65 levels in the nuclear fraction after CRAT downregulation (Figure 5d). Phosphorylated IĸBα induces its proteasomal degradation, leading to decreased total IĸBα and liberation of p65, enabling shuttling of p65 into the nucleus (Giridharan & Srinivasan, 2018). CRAT silencing induced increased phosphorylation of IĸBα and decreased total IĸBα level in the cytoplasm as well as increased p65 level in the nucleus due to nuclear‐cytoplasmic shuttling (Figure 5d), which led to transcriptional activation of SASPs by the NF‐ĸB pathway (Israel, 2010). In addition, knockdown of p65, which is an essential NF‐ĸB component, significantly inhibited the mRNA expression of SASP genes, the secretion of SASPs, and the increase in SA‐β‐gal‐positive cells induced by CRAT downregulation (Figure 5e; Figures S4 and S5a,b), suggesting that NF‐ĸB is the regulator of SASPs induced by CRAT downregulation.

SASPs are known to be activated by NF‐ĸB or C/EBPβ at the transcription level; hence, we cannot rule out the possibility that C/EBPβ may also activate SASPs (Kumari & Jat, 2021). Hence, we determined to what extent NF‐ĸB and C/EBPβ regulate activation of SASPs after CRAT knockdown by comparing the effect of NF‐ĸB and C/EBPβ downregulation on CRAT knockdown‐induced SASPs. Knockdown of p65 simultaneously with CRAT knockdown almost completely inhibited the activation of SASPs (Figure 5e), while C/EBPβ knockdown only partially blocked the increased SASPs induced by CRAT suppression (Figure S6). Hence, we confirmed that NF‐ĸB activated by cGAS‐STING pathway is the primary regulator of the SASP induced by CRAT downregulation.

### 3.6 Fibroblast‐specific CRAT‐knockout mice show senescence phenotypes

**FIGURE 6:**
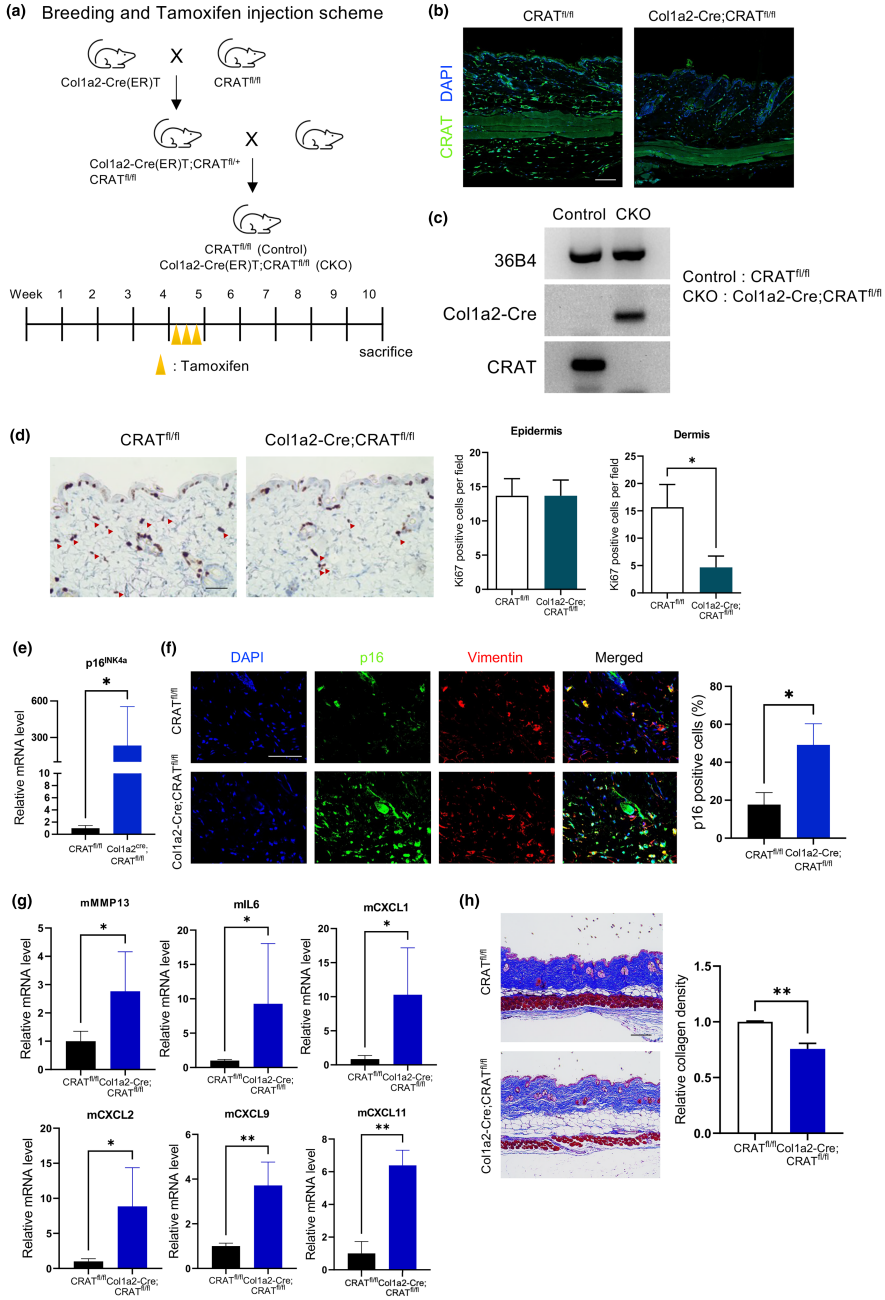
Fibroblast‐specific Carnitine acetyltransferase (*CRAT)* knockout mice show senescent phenotypes. (a) CRAT^fl/fl^ mice were crossed with a tamoxifen‐inducible Col1a2‐Cre recombinase (Col1a2‐Cre(ER)T) for generation of fibroblast‐specific CRAT‐knockout mice. Four‐week‐old mice were subjected to tamoxifen injection (40 mg/kg) three times in a week, which induced fibroblast‐restricted Cre expression, and then the mice were sacrificed at Week 10 for analysis. (b) Immunofluorescence staining was performed using paraffin‐embedded back skin tissues of control and conditional knockout mice obtained at Week 10 to verify reduced expression of CRAT specifically in the dermis. *Scale bar* = 100 μm. (c) CRAT deletion in the fibroblasts was verified by PCR analysis of RNA extracts from primary fibroblasts, isolated from the dermis of the back skin at Week 10. Col1a2‐Cre was used as a positive or negative control, and 36B4 was used as a loading control. (d) Ki67 staining was performed by immunohistochemistry to examine proliferating cells. Ki67‐positive cells expressed at the epidermis or dermis were counted per image field (*n* = 3 CRAT^fl/fl^, *n* = 3 Col1a2‐Cre;CRAT^fl/fl^). *Scale bar* = 100 μm. Representative images are shown. (e) p16 mRNA level in the indicated mice skin, normalized to GAPDH level. (f) Representative images of immunofluorescence staining of p16 (green) and vimentin (red) in the skin of indicated mice, and quantification of p16‐ and vimentin‐positive cells among 4′,6‐diamidino‐2‐phenylindole (DAPI)‐positive cells (*n* = 3 CRAT^fl/fl^, *n* = 3 Col1a2‐Cre;CRAT^fl/fl^). *Scale bar* = 50 μm. (g) The mRNA levels of cytokines were measured by quantitative real time‐PCR using back skin tissue of control and fibroblast‐specific CRAT‐knockout mice, normalized to GAPDH levels. (h) Back skin sections were stained with Masson's trichrome staining. Collagen density was quantified using ImageJ by color convolution plugin. *Scale bar* = 100 μm. Representative images are shown (*n* = 5 CRAT^fl/fl^, *n* = 5 Col1a2‐Cre;CRAT^fl/fl^). Data are shown as mean ± standard error of mean (SEM). **p < 0.01, *p < 0.05 versus CRAT^fl/fl^ analyzed by *t* test.

Consistent with human skin, CRAT expression was decreased in old (24‐month‐old) wild‐type C57B6/J mice compared with young (6‐month‐old) mice (Figure S7). To confirm that CRAT deficiency in fibroblasts induces senescence phenotypes in vivo, we used fibroblast‐specific CRAT‐knockout mice. By breeding CRAT flox/flox mice and tamoxifen‐inducible Col1a2‐Cre mice, we generated Cola2‐Cre‐(ER)T;CRAT^fl/fl^ mice, which were subjected to tamoxifen injection to induce Cre recombinase expression (Figure 6a). The knockout efficiency of CRAT‐knockout mice was confirmed by immunofluorescence staining of the skin tissue and PCR analysis of cultured fibroblasts obtained from the skin of wild‐type and knockout mice after tamoxifen administration (Figure 6b,c).

We examined senescence phenotypes such as cell proliferation, p16 expression, and SASPs in fibroblast‐specific CRAT‐knockout mice. Decreased proliferation was determined by the significantly reduced number of Ki67‐positive cells in the dermis of fibroblast‐specific CRAT‐knockout mice compared to that in control mice, while the number of Ki67‐positive cells was relatively constant in the epidermis. (Figure 6d). Moreover, fibroblast‐specific CRAT‐knockout mice showed increased mRNA and protein expression of p16, which is the in vivo senescence marker (Figure 6e,f). To confirm the increased p16 expression in fibroblasts, we quantified the number of cells positive for both p16 and the fibroblast marker vimentin following co‐staining of the two markers (Figure 6f). Increased expression of SASPs, including *MMP13*, *IL6*, *CXCL1*, *CXCL2*, *CXCL9*, and *CXCL11* was also observed in fibroblast‐specific CRAT‐knockout mice (Figure 6g). Interestingly, collagen density, as measured by Masson's trichrome staining, was significantly decreased in fibroblast‐specific CRAT‐knockout mice (Figure 6h). Overall, these findings demonstrate that CRAT deficiency in fibroblasts resulted in the senescent phenotypes of mouse skin.

## 4 DISCUSSION

The present study showed that CRAT deficiency contributes to skin aging by causing mitochondrial dysfunction‐induced cellular senescence. We performed RNA sequencing of young and aged individuals and identified CRAT, which was significantly downregulated in aged individuals, as a key regulator of aging. Previous studies have reported the significant role of CRAT in metabolic contexts such as obesity, diabetes, calorie restriction, and exercise in tissues with high mitochondrial content and high energy demands, such as muscle and liver (Davies et al., 2016; Mezhnina et al., 2020; Muoio et al., 2012; Seiler et al., 2014, 2015); yet, no studies have elucidated the role of CRAT in senescence.

We found that CRAT silencing in fibroblasts reproduced cellular senescence phenotypes, including decreased proliferation, enhanced SA‐β‐gal activity, and SASP production by inducing oxidative stress and morphological changes in mitochondria. Mitochondrial dysfunction is known to induce SASPs and senescence (Correia‐Melo et al., 2016; Riley & Tait, 2020; van der Rijt et al., 2020), but the drivers of mitochondrial dysfunction and cellular senescence are not well‐defined. Previous studies on cellular senescence have focused mainly on DNA damage responses that regulate p16^INK4a^, p21^Cip1^, or ATM (Kumari & Jat, 2021) but mitochondria are also crucial for senescence induction. As CRAT is a mitochondrial matrix enzyme, we focused on mitochondrial oxidative stress and morphology in CRAT‐knockdown HDFs. Indeed, CRAT silencing in HDFs led to mitochondria‐specific ROS production, mitochondrial morphological alterations, and SASP production, all of which were mitigated by NAC treatment. CRAT regulates acetyl‐CoA balance and fatty acid substrate utilization in the mitochondria (Muoio et al., 2012); hence, mitochondrial ROS generation by CRAT knockdown might be attributed to the accumulation of fatty acids (Schönfeld & Wojtczak, 2008).

Mitochondrial damage is also accompanied by altered mitochondrial metabolism in aging and age‐associated diseases (López‐Lluch et al., 2018); hence, we investigated the effect of CRAT on cellular bioenergetics. CRAT silencing in HDFs was associated with metabolic inflexibility, characterized by reduced maximal respiration and spare respiratory capacities in the mitochondria. Decreased maximum respiration and spare respiratory capacities indicate that CRAT‐knockdown cells are susceptible to mitochondrial stress due to reduced metabolic flexibility, which refers to the ability of cells to adapt to changes in energy demands through rapid oxidation to meet metabolic challenges. Reduced mitochondrial respiratory capacity during aging has been previously reported in skeletal muscles and monocytes (Porter et al., 2015). In contrast, basal and compensatory glycolysis was significantly increased upon CRAT knockdown. Overall, CRAT silencing resulted in significant changes in cellular bioenergetics, a metabolic shift from oxidative phosphorylation to glycolysis, implying that pyruvate is mainly converted to lactate instead of being transported into the mitochondria for conversion to acetyl‐CoA (Sabbatinelli et al., 2019). This is in line with the results of previous studies which showed that aging impaired oxidative phosphorylation activity but increased glycolysis as a compensatory metabolic pathway in aged cells and tissues (Cho et al., 2017; Feng et al., 2016; Korolchuk et al., 2017; Oblong et al., 2020).

Here, we uncovered the novel role of CRAT in aging which connects mitochondrial dysfunction and cellular senescence by mitochondrial damage‐induced mtDNA release and cGAS‐STING‐NF‐κB pathway activation. NF‐κB is a well‐known transcription factor that induces the expression of SASPs (Songkiatisak et al., 2022). Several recent studies have indicated that mtDNA induces senescence by activating cGAS‐STING and subsequently stimulating NF‐κB nuclear translocation (Choi et al., 2018; Dou et al., 2017; Gao et al., 2020; Gluck et al., 2017; Hu et al., 2022). Moreover, mouse embryonic fibroblasts from cGAS‐knockout mice showed reduced senescence phenotypes, suggesting that cGAS is essential for cellular senescence induction (Yang et al., 2017). In this regard, we uncovered the molecular mechanism by which CRAT silencing released mtDNA into the cytosol, leading to cGAS‐STING‐NF‐κB activation to initiate SASP secretion and senescence phenotypes.

These results provide insights into how CRAT regulates senescence in the skin in vivo; fibroblast‐specific CRAT knockout mice showed senescent phenotypes and increased SASP secretion in the skin. Specifically, fibroblast‐specific loss of CRAT activity in vivo led to reduced proliferation of fibroblasts, enhanced expression of SASPs, and decreased dermal collagen density. As senescent cells exert detrimental effects on neighboring cells through the secretion of SASPs in a paracrine manner, fibroblast‐specific CRAT deficiency may contribute to inflammaging by increasing inflammation during organismal aging. Skin aging is characterized by ECM deficiency and increased expression of MMPs (Freitas‐Rodríguez et al., 2017), which belong to SASPs. Fibroblast‐specific CRAT‐knockout mice showed significantly decreased collagen density, possibly owing to ECM degradation by increased MMPs, which remains to be elucidated in future studies. Additionally, the impact of administrating antioxidants such as NAC to CRAT knockout mice could be further investigated to determine whether senescence phenotypes are mitigated by alleviating mitochondrial oxidative stress.

Further studies on the regulatory mechanisms by which CRAT level is reduced during aging may enable the prevention or reversal of CRAT deficiency. A previous report on age‐related DNA methylation changes in blood revealed that CRAT is highly methylated, suggesting that CRAT expression may be reduced during aging due to increased methylation (Madrigano et al., 2012). In addition, calorie restriction, which is known to attenuate organismal aging, leads to an increased expression of CRAT and its corresponding fatty acid oxidation products in the mouse liver (Mezhnina et al., 2020). These results suggest that epigenetic regulation and calorie restriction may be effective in regulating CRAT expression to combat aging.

Taken together, our results demonstrate that CRAT is a key regulator of mitochondrial dysfunction‐induced cellular senescence in dermal fibroblasts. CRAT silencing causes mitochondrial dysfunction, inflammation, and senescence through activation of the cGAS‐STING and NF‐ĸB pathways. Hence, prevention or reversal of CRAT deficiency may provide a novel therapeutic approach for aging and age‐related disorders.

## AUTHOR CONTRIBUTIONS

Study conception and design and draft manuscript preparation: Min Ji Song, Chi‐Hyun Park, Dong Hun Lee, and Jin Ho Chung; data collection: Min Ji Song; analysis and interpretation of results: Min Ji Song, Chi‐Hyun Park, Haesoo Kim, and Sangbum Han. All authors reviewed the results and approved the final version of the manuscript.

## FUNDING INFORMATION

This work was supported by a National Research Foundation of Korea (NRF) grant funded by the Korean government (MSIT) (No. 2019R1F1A1059005) and the Seoul National University Hospital Research Fund (04‐2016‐0250).

## CONFLICT OF INTEREST STATEMENT

The authors declare that they have no competing interests.

## Supporting information


Figure S1–Figure S7.

## Data Availability

The data that support the findings of this study are available on request from the corresponding author. The data are not publicly available due to privacy or ethical restrictions.
