# Supplementary material for: Carnitine acetyltransferase deficiency mediates mitochondrial dysfunction‐induced cellular senescence in dermal fibroblasts
Source: Aging Cell. 2023 Oct 13;22(11):e14000. doi: 10.1111/acel.14000 (PMC10652321; doi:10.1111/acel.14000)
Supplement: Supplementary file 1 — Figure S1–Figure S7. [file ACEL-22-e14000-s001.pdf]

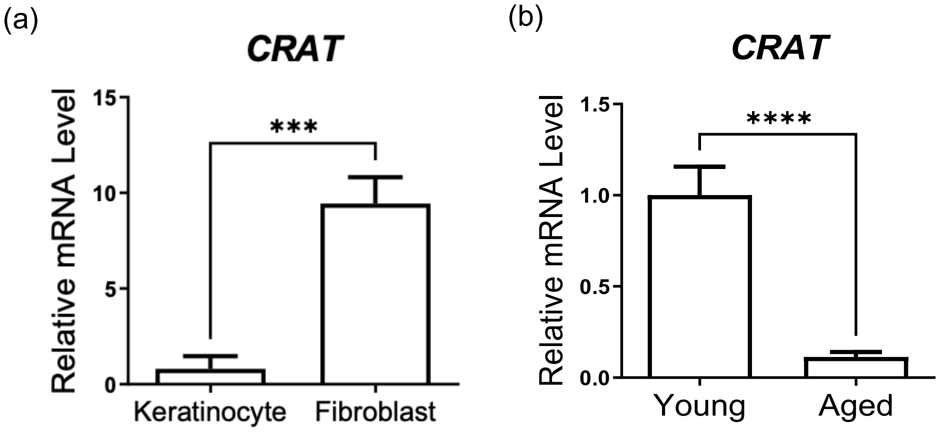

Supplementary Figure S1. CRAT expression is relatively high in dermal fibroblasts and downregulated in the aged dermal fibroblasts. (a) Relative expression level of CRAT in primary cultured epidermal keratinocytes and dermal fibroblasts was measured by RT-PCR (n = 3-4). (b) CRAT mRNA expression level of cultured primary fibroblasts from the young (n = 6) and the aged (n = 6) skin. Data represent the mean ± standard error of mean (SEM). ****p < 0.0001, ***p < 0.001 versus Keratinocyte or Young analyzed by t test.

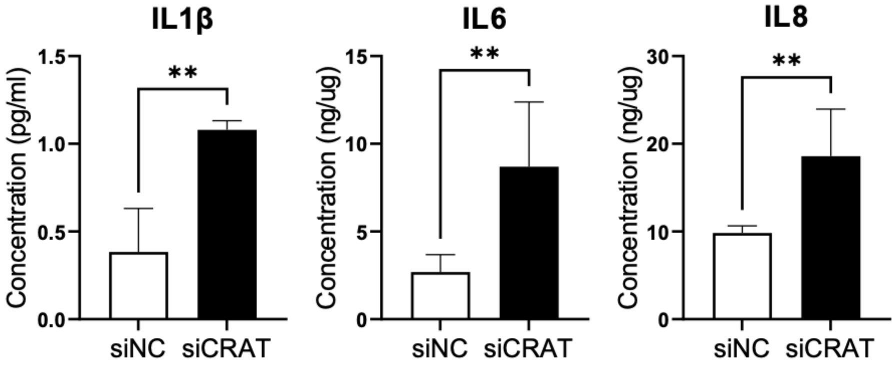

Supplementary Figure S2. Protein level of secreted SASPs after CRAT knockdown. The concentration of *IL1β, IL6,* and *IL8* secreted in the media after CRAT knockdown in dermal fibroblasts was measured by cytokine analysis. Data represent the mean ± standard error of mean (SEM). $^{**}p < 0.01$ versus siNC analyzed by *t* test.

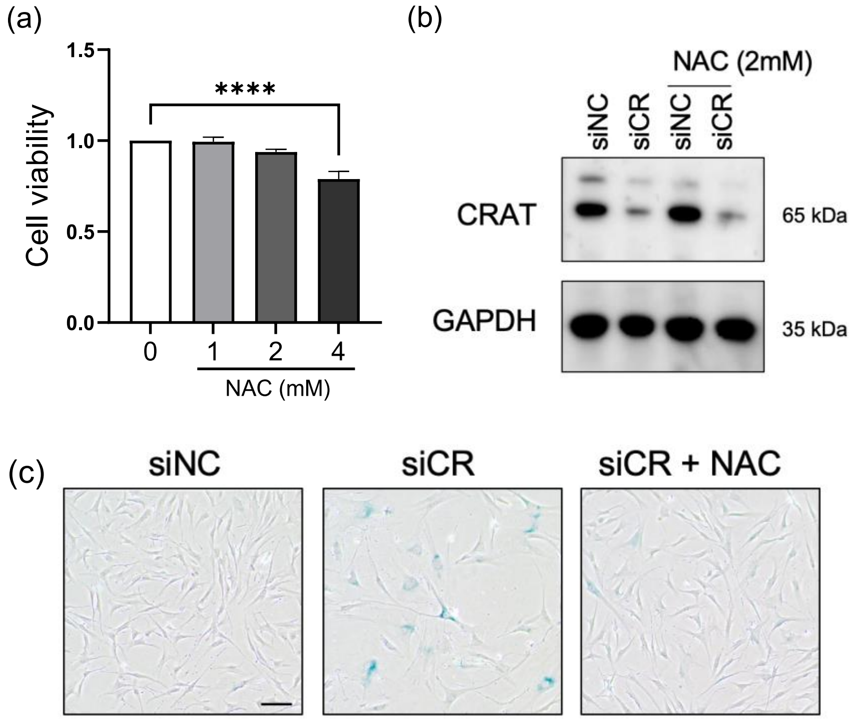

Supplementary Figure S3. The effect of NAC treatment on cell viability, CRAT protein expression, and CRAT knockdown-induced senescence. (a) Cell viability was measured by WST-8 assay after treatment of NAC at indicated concentrations (1, 2, and 4 mM). (b) HDFs are transfected with siNC and siCRAT and simultaneously treated with NAC (2 mM). CRAT protein expression was analyzed by western blotting. (c) HDFs were transfected with siRNAs in the presence or absence of NAC (2 mM) for 48 h. Cells were subjected to senescence-associated-β-galactosidase (SA-β-gal) staining 10 days after transfection. *Scale bar* = 100 μm. Data represent the mean ± standard error of mean (SEM). ****$p < 0.0001$ versus control analyzed by *t* test.

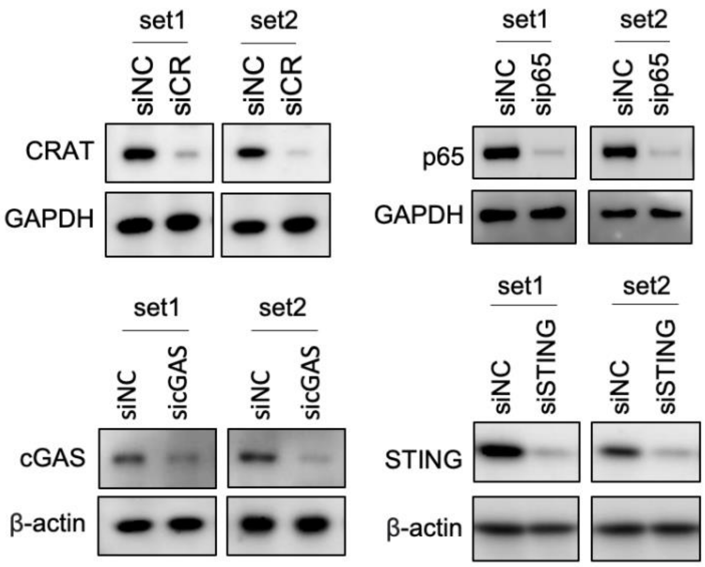

Supplementary Figure S4. Knockdown efficiency of siRNA-mediated CRAT, p65, cGAS, and STING knockdown. Western blotting was performed to confirm siRNA-mediated knockdown of CRAT, p65, cGAS, and STING at protein level.

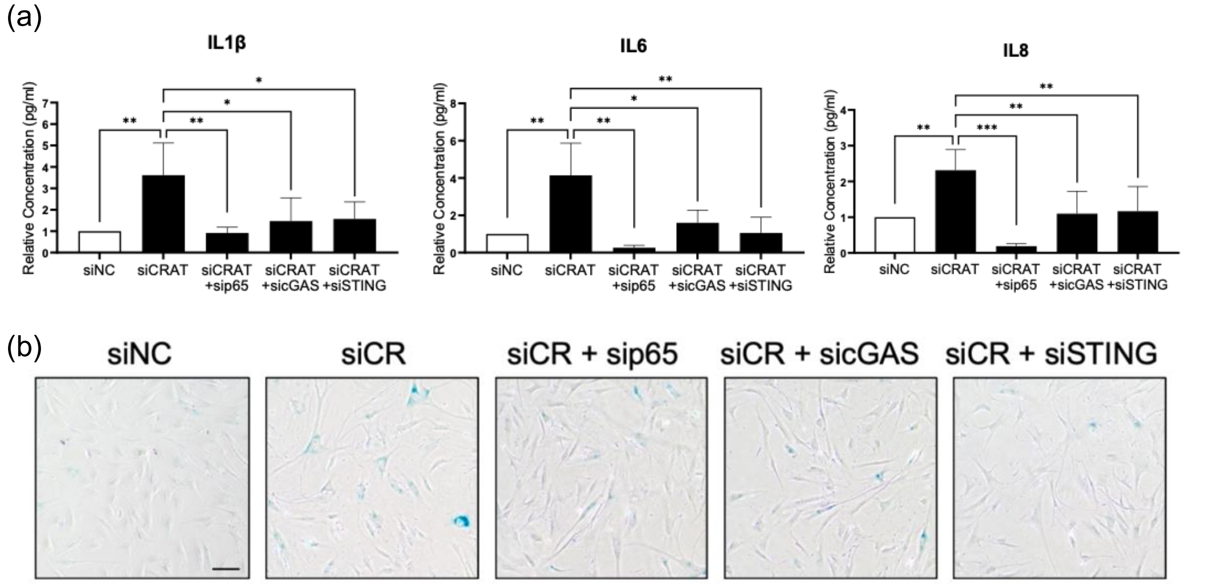

Supplementary Figure S5. Cellular senescence induced by CRAT knockdown is inhibited by double-knockdown of CRAT with p65, cGAS, or STING. (a) Relative concentration of *IL1β, IL6,* and *IL8* secreted in the media after siRNA-mediated knockdown of CRAT or double knockdown of CRAT with p65, cGAS, or STING in dermal fibroblasts was measured by cytokine analysis. ***$p < 0.001$, **$p < 0.01$, *$p < 0.05$ versus siCR by analysis of variance (ANOVA). (b) SA-β-gal staining was conducted 10 days after co-transfection of siNC or siCRAT without or with sip65, sicGAS, or siSTING. *Scale bar* = 100 μm.

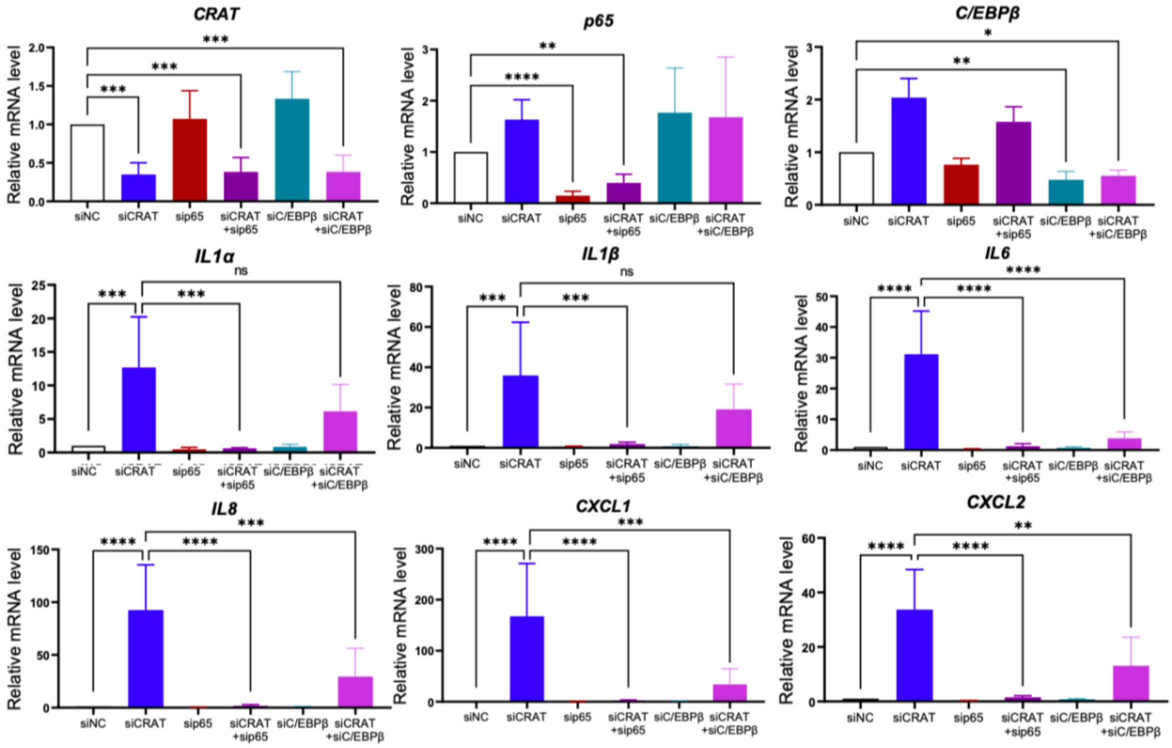

Supplementary Figure S6. Increased SASP mRNA expression level by CRAT knockdown is inhibited by double-knockdown with p65 or C/EBPβ. HDFs were subjected to transient transfection for 48 h with siNC, siCRAT, sip65, or siC/EBPβ or co-transfection of siCRAT with sip65 or siC/EBPβ. mRNA expression of SASP genes were analyzed by RT-PCR three days after media change (n = 4). Data shown as mean ± standard error of mean (SEM), ****$p < 0.0001$, ***$p < 0.001$, **$p < 0.01$ versus siNC or siCRAT by analysis of variance (ANOVA).

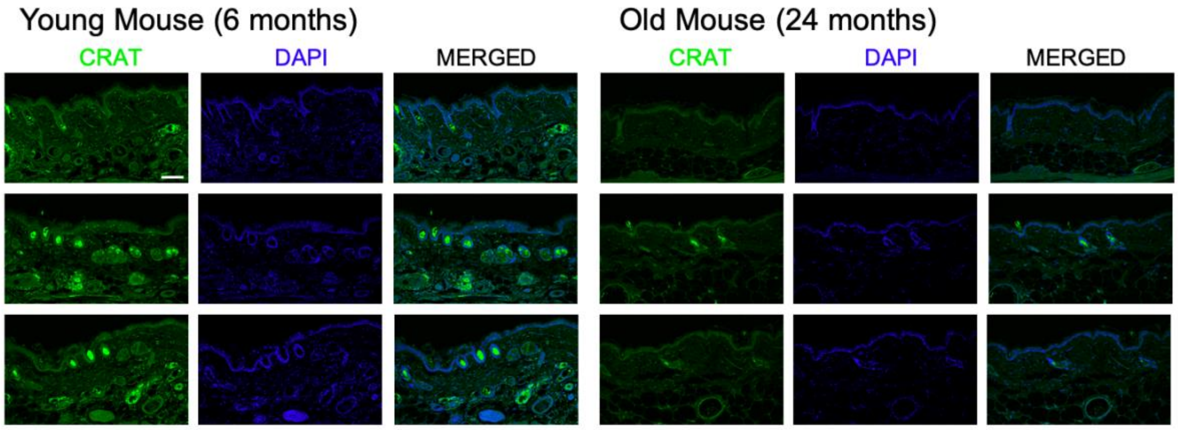

Supplementary Figure S7. Immunofluorescence staining of CRAT in young and old mice. Immunofluorescence staining of CRAT was conducted in young (6 months old) and old (24 months old) wild-type C57B6/J mice. *Scale bar* = 100 μm.
